# Intraneural vascularity of the median, ulnar and common peroneal nerve: Microvascular ultrasound and pathophysiological implications

**DOI:** 10.1002/ajum.12334

**Published:** 2023-02-17

**Authors:** Johannes Deeg, Felix Mündel, Alexander Loizides, Leonhard Gruber, Hannes Gruber

**Affiliations:** ^1^ Department of Radiology Medical University Innsbruck Anichstraße 35 6020 Innsbruck Austria

**Keywords:** high‐resolution ultrasound, intraneural vascularity, musculoskeletal ultrasound, peripheral neuropathy, superb microvascular imaging

## Abstract

**Objectives:**

Changes in the microvascular environment are considered crucial in the pathogenesis of compression neuropathies. Several studies have demonstrated elevated intraneural vascularity in severe neuropathy compared with healthy subjects, where intraneural vascularity is considered predominantly undetectable. The aim of this study was to assess and quantify intraneural vasculature by superb microvascular imaging (SMI) in healthy volunteers in the median, ulnar and common peroneal nerve.

**Methods:**

Intraneural vascularity was quantified in 26 healthy volunteers (312 segments overall) by SMI sonography using a 22‐MHz linear transducer. Individual nerve segment vascularity was compared with the mean vascularity using one‐way ANOVA and Kruskal–Wallis tests, respectively. Vendor‐provided quantification and manual vessel count were compared by linear regression analysis.

**Results:**

Intraneural vascularity was detectable in all nerve segments (100.0%). Vessel density was highest in the median nerve at the wrist (1.54 ± 0.44/mm^2^, P < 0.0001) and lowest in the sulcal ulnar nerve (0.90 ± 0.34/mm^2^, P < 0.0001). Vendor‐provided automated quantification severely overestimated vascular content compared with manual quantification.

**Conclusion:**

Superb microvascular imaging can facilitate the visualisation of nerve vascularity and even detect local variations in vessel density. The pathophysiological implications for peripheral neuropathies, especially compression neuropathies, warrant further investigation, but the absence of visible intraneural vasculature as a negative finding in the diagnostic of compression neuropathies should be interpreted with caution, as the intraneural vascularity may lie beyond the 18 MHz resolution power of a transducer.

**Abbreviations**
CTS: carpal tunnel syndrome
HIF: hypoxia‐inducible factors
HRUS: high‐resolution ultrasound
SMI: superb microvascular imaging
SNUS: sulcus nervi ulnaris syndrome
VEGF: vascular endothelial growth factor

## Introduction

Over the past decades, ultrasonography has received increasing attention in the evaluation of musculoskeletal diseases, becoming now the first‐line imaging diagnostic modality for nerve entrapment neuropathies.^1^ These advances were – among other findings – facilitated by innovations regarding imaging hardware, software algorithms, faster data processing and new sonographic applications like elastography.

Superb microvascular imaging (SMI) itself is a modified Doppler algorithm enabling the visualisation of smaller vessels with slower blood flow compared with existing Doppler modalities.^2^ SMI is currently being used for many diagnostic regions including musculoskeletal applications.^3^ As it still remains unclear how far or in which way microvascularity is changed in compression neuropathies, SMI may be added as a new diagnostic tool for evaluating peripheral compression syndromes.

As changes in the microvascular environment are considered crucial in the pathogenesis of compression neuropathies,^4^ over the past decades, evaluation of intraneural vascularity in cases of entrapment neuropathies has been attempted by means of colour‐coded Doppler ultrasound (CDUS),^5^ or power Doppler (PDUS),^6^, ^7^ and more recently, by means of SMI.^8^, ^9^

Many studies have tried to uncover the exact pathophysiologic mechanism of repeated compression of neural structures.^4^, ^10^ Complex pathophysiologic changes are initiated following impaired blood supply due to some kind of compression of the affected nerve.^11^ Altered blood supply leads to a breakdown of the blood–nerve barrier, which in turn results in oedematous swelling of the nerve and microvascular changes.^12^, ^13^ Furthermore, the impaired blood supply leads to an upregulation of hypoxia‐inducible factors (HIF), which in turn induces an upregulation of vascular endothelial growth factor (VEGF).^14^ VEGF is known to have a neuroprotective and an angioproliferative effect, resulting in an increase in the microvessel density in experimental animal studies.^15^, ^16^

In one of the first studies that investigated nerve vascularity in the setting of carpal tunnel syndrome (CTS), Mallouhi *et al*.^5^ suggested the presence of intraneural hypervascularisation as the feature of highest accuracy among all sonography criteria for diagnosing CTS. Since then, many other studies investigated the diagnostic value of intraneural vascularity in cases of entrapment neuropathies and suggested increased intraneural vascularity as a good diagnostic criterion.^6^, ^7^, ^17^

Intraneural vascularity may be inaccessible to conventional Doppler methods due to their small diameters and slow blood flow. To detect those tiny vessels, Chen *et al*.^8^ evaluated the intraneural vascularity in 50 CTS patients and 25 healthy subjects by SMI. Compared with CDUS and PDUS, SMI was more accurate in the detection of intraneural blood flow in patients with CTS, yet even in SMI 13 of the 25 healthy subjects showed no visible intraneural vascularisation. Similar results regarding vessel sensitivity in CTS were reported by Karahan *et al*.^9^ and Yildiran *et al*.^18^ who also reported a correlation between SMI grading and electrophysiology results.

Several other studies focussed on other nerves such as the ulnar nerve. Ghanei et al.^19^ and Cheng *et al*.^20^ showed an increased intraneural vascularity in cases of sulcus nervi ulnaris syndrome (SNUS) compared with a healthy control group. Again only three of 44 healthy subjects in the study of Ghanei and three of 50 in the study of Cheng showed a detectable intraneural vascularity.

In this study, we examined the intraneural vascularity of the median, ulnar and common peroneal nerve in healthy volunteers at typical anatomic locations for nerve entrapment neuropathies using a 22‐MHz linear transducer with SMI and tried to answer whether intraneural vascularity in healthy subjects can really be detected in just a minority of cases.

## Materials and methods

### Healthy volunteers

Twenty‐six participants were examined in this study. Medical ethics committee (committee vote 1320/2019), and written informed consent was obtained from each subject prior to the investigation.

All subjects had no known prior injury at the median, ulnar and common peroneal nerve. Further exclusion criteria were predisposing conditions for polyneuropathy such as diabetes, hypo‐ or hyper‐thyroidism and pregnancy.

### Sonographic examination

Sonographic examinations were performed using an Aplio i900 ultrasound machine (Canon Medical Systems, Otawara, Japan) with a 22‐MHz linear‐array transducer (i22LH8) with the predefined pre‐sets: PRF (1,2), mechanical index (0,8), B‐picture frequency (19 MHz) and Doppler frequency (10 MHz). No filter or beam steering was used. The predefined median, ulnar and common peroneal nerve segments were scanned by two experienced radiologists with at least 10 years of experience in musculoskeletal ultrasound.

The median nerve was measured with the subject sitting with the elbow flexed to 120° and the hand in a supine position. The first segment (proximal MN) was 12 cm proximal to the crease of the wrist and the second segment (distal MN) at the carpal tunnel entrance.

The ulnar nerve was measured with the subject sitting with the elbow only slightly flexed and the hand in a supine position. The first segment (proximal UN) was 2–3 cm proximal to the medial humeral epicondyle; the second segment (distal UN) was scanned within the cubital tunnel while visualising the bony olecranon and medial humeral epicondyle.

The common peroneal nerve was measured with the subject lying in the left/right lateral position with the knee slightly flexed. The first segment (PN prox) was the edge of the biceps femoris muscle and the fibula head and the second segment (PN dist) distal to the fibula head.

**Figure 1:**
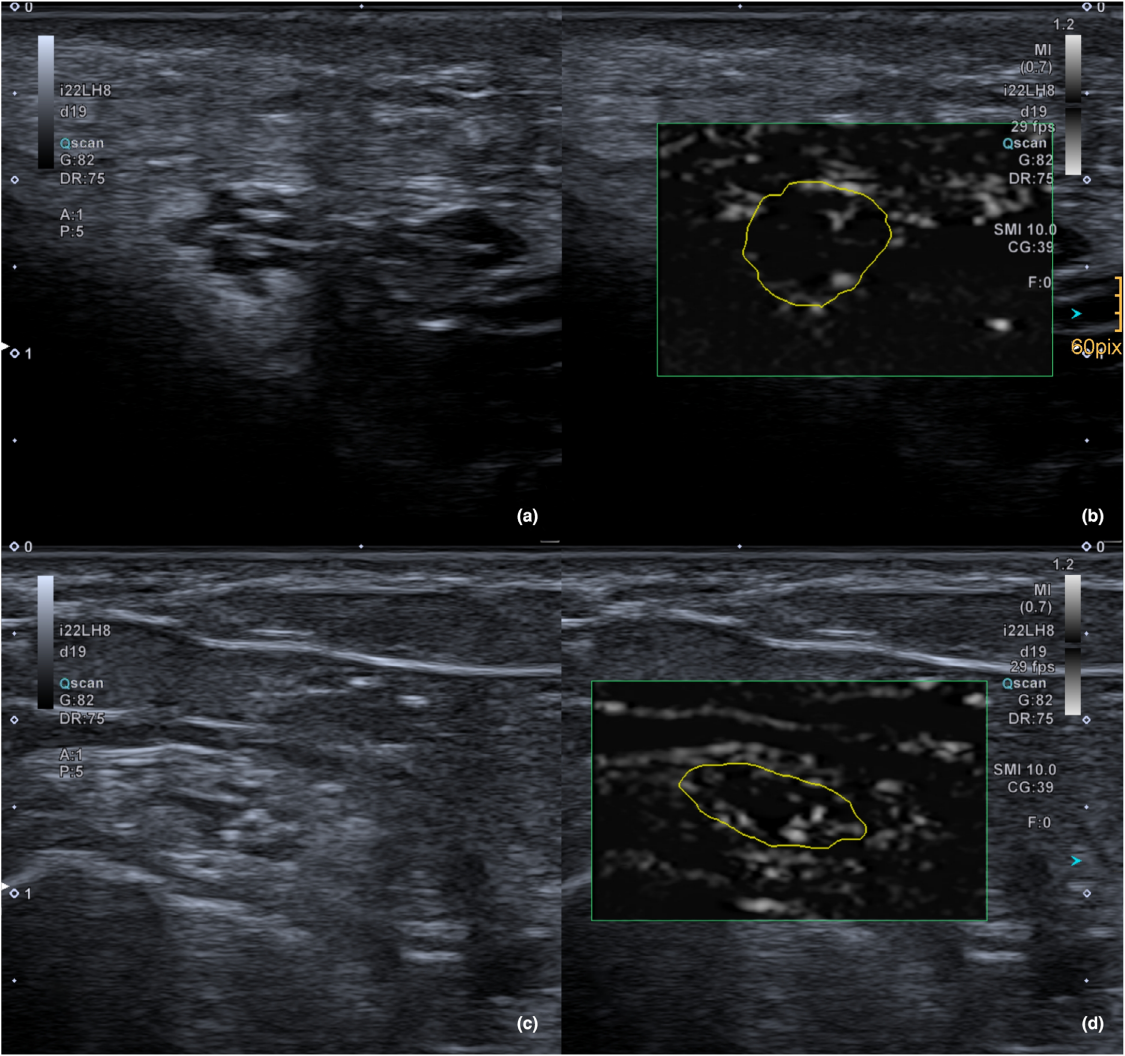
Illustration of the ulnar nerve (a, b) and common peroneal nerve (c, d) with a normal b‐mode transversal scan on the left and the SMI mode overlay on the right. In both nerves, the intraneural vascularity is clearly visible. SMI, superb microvascular imaging.

After depicting the nerve in the axial plane, vascularity was assessed by the SMI mode (see Figure 1). Using a cine‐loop to define the systolic phase, a freehand region of interest (ROI) was drawn along the neural contour to generate a vendor‐proprietary vascular index (SMI_perc_), describing the ratio of pixels considered vasculature within a given ROI.

After conclusion of the ultrasound examinations, a manual vessel count was performed for each nerve segment and the vessel density was calculated by dividing the vessel number through the segment's cross‐sectional area (CSA); results are given as vessels per square millimetre (n/mm^2^).

### Statistics

The collected data were stored in Microsoft Excel 16.16.24 (Microsoft, Redmond, WA, USA). Statistical software used was GraphPad Prism 8.4.3 (GraphPad Software LLC, La Jolla, CA, USA) and SPSS 26.0 for Windows (SPSS, Chicago, IL, USA). Demographic parameters: age, sex, height, weight, BMI and right or left hand dominance are presented descriptively. Software‐based SMI vessel density calculation (SMI_perc_), manually counted vessels and vessel density (vessel count n per nerve segment CSA in mm^2^) for each nerve segment were compared with the averaged findings of all nerve segments, which served as control. For SMI_perc_, an ordinary one‐way ANOVA with a Holm–Sidak correction for multiple testing was used, and for both absolute and normalised vessel count (vessel density), a Kruskal–Wallis test with Dunn's correction due to its non‐Gaussian distribution was used. A linear regression was performed to assess the correlation between the vendor‐provided measurements and the manual vessel count. A linear regression analysis with bootstrapping (10.000 samples, bias‐corrected accelerated [BCa] 95% confidence intervals [CIs]) was carried out to evaluate the influence of the predictors age, sex, height, weight, BMI, hand dominance, neural depth, CSA and ratio of distal‐to‐proximal nerve segment on vessel density in all nerve segments. Results are given as B‐value, standard error (SE), P‐value and BCa 95% CIs.

### Ethics approval

The study was approved by the local medical ethics committee (final decision of the ethics committee 1320/2019). We confirm that we have read the Journal's position on issues involved in ethical publication and affirm that this report is consistent with those guidelines.

## Results

### Demographics

Overall, 26 participants and 156 nerve segments for each side were examined. Fifteen participants were female (57.7%), and the overall age was 28.5 ± 5.4 years (range 20–41 years). The average height, weight and BMI of participants were 172.7 ± 8.5 cm (range 155–191 cm), 67.8 ± 12.5 kg (range 50–104 kg) and 22.6 ± 2.5 kg/m^2^ (range 17.9–28.5 kg/m^2^) respectively. The majority were right‐handed (*n* = 24, 92.3%).

### SMI_perc_ , absolute vessel counts and vessel density of nerve segments

**Figure 2:**
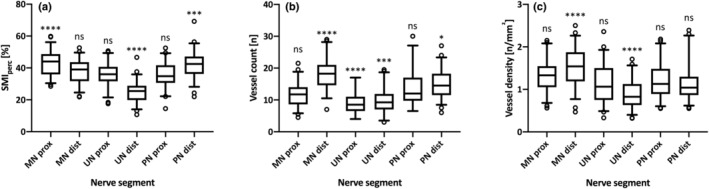
Comparison of average SMI_perc_ (a), absolute vessel count (b) and vessel density (c) for all examined nerve segments against the average values for all nerves. MN prox, median nerve proximal; MN dist, median nerve distal; ns, not significant; PN prox, common peroneal nerve proximal; PN dist, common peroneal nerve distal; SMI, superb microvascular imaging; UN prox, ulnar nerve proximal; UN dist, ulnar nerve distal.

Intraneural vascularity was detected in all nerve segments (*n* = 312, 100.0%). Software SMI vessel density calculation (SMI_perc_), manual vessel count and vessel density showed a significant difference between various nerve segments in regard to SMI_perc_ as well as absolute vessel count and vessel density (Table 1). Most notably, vessel density was significantly higher in the distal median nerve (1.54 ± 0.44, P < 0.0001) but significantly lower in the distal ulnar nerve (0.90 ± 0.34, P < 0.0001) compared with the average vessel density across all nerve segments (Figure 2).

**Table 1:** Average nerve segment SMI_perc_, absolute and relative vessel count (compared to proximal median nerve)

Nerve segment	SMI_perc_ (%)	P‐value^§^	Vessel count (*n*)	P‐value*	Vessel density (n/mm^2^)	P‐value*
Median nerve, proximal	43.21 ± 7.71	<0.0001	11.69 ± 3.73	>0.9999	1.33 ± 0.38	0.1981
Median nerve, distal	37.81 ± 7.46	0.7555	18.41 ± 4.98	<0.0001	1.54 ± 0.44	<0.0001
Ulnar nerve, proximal	35.98 ± 7.54	0.7933	9.41 ± 3.55	<0.0001	1.12 ± 0.47	>0.9999
Ulnar nerve, distal	25.00 ± 6.64	<0.0001	9.84 ± 4.38	0.0002	0.90 ± 0.34	<0.0001
Peroneal nerve, proximal	35.86 ± 7.90	0.7933	13.98 ± 5.81	>0.9999	1.21 ± 0.43	>0.9999
Peroneal nerve, distal	42.05 ± 8.51	0.0002	15.06 ± 4.59	0.0246	1.16 ± 0.44	>0.9999

SMI, superb microvascular imaging.

^§^ Ordinary one‐way ANOVA.

* Kruskal–Wallis test.

### Correlation between SMI_perc_ and vessel count

**Figure 3:**
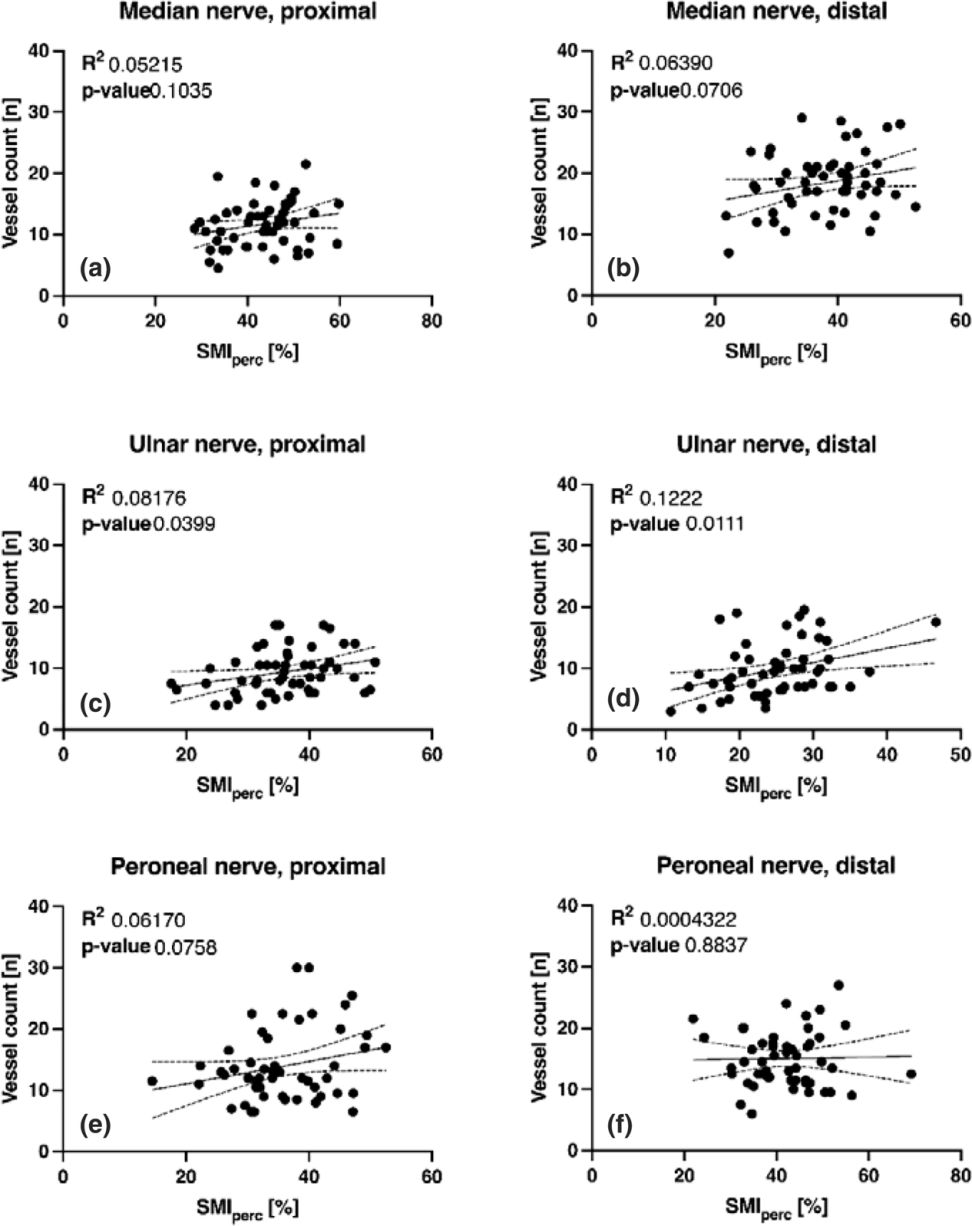
Correlation between ROI‐based SMI quantification (SMI_perc_) and manual vessel count for the proximal median nerve (a), distal median nerve (b), proximal ulnar nerve (c), distal ulnar nerve (d), proximal peroneal nerve (e) and distal peroneal nerve (f). ROI, region of interest; SMI, superb microvascular imaging.

Overall, only the proximal ulnar (slope 0.135 ± 0.064, *R* ^2^ 0.082, P = 0.040, Figure 3c) and distal ulnar nerve (slope 0.231 ± 0.087, *R* ^2^ 0.122, P = 0.011, Figure 3d) showed a weak correlation between SMI_perc_ and manual vessel count with all other nerve segments exhibiting virtually no correlation between these two measurements (Figure 3).

### Parameters influencing vessel count

Several factors were associated with an increase in vessel density, yet at varying degrees of impact (Table 2). The strongest predictors with a positive correlation were right‐handedness (*B* = 0.349 [95% CI 0.205–0.488], P < 0.0001) and ratio (*B* = 0.139 [95% CI 0.006–0.278], P = 0.038). Several predictors had a significant, yet minimal impact; among them, age with a positive correlation (P = 0.002); and nerve depth (P = 0.002) and CSA (P < 0.0001) with a negative impact. Sex, height, weight and BMI showed no significant influence.

**Table 2:** Linear regression results of factors influencing vessel density

	*B*	SE	P‐value	BCa 95% confidence interval	
				Lower	Upper
Sex (male)	0.013	0.086	0.885	−0.156	0.179
Age (years)	0.015	0.005	0.002	0.006	0.024
Height (cm)	0.006	0.023	0.782	−0.041	0.056
Weight (kg)	−0.008	0.029	0.77	−0.065	0.045
BMI (kg/m^2^)	0.03	0.096	0.746	−0.157	0.238
Hand Dominance (right)	0.349	0.07	<0.0001	0.205	0.488
Nerve depth (mm)	−0.036	0.012	0.002	−0.061	−0.018
CSA (mm^2^)	−0.031	0.007	<0.0001	−0.044	−0.017
Ratio	0.139	0.067	0.038	0.006	0.278

BMI, Body mass index; CSA, cross‐sectional area.

## Discussion

The assessment of intraneural vascularity and its role in the pathogenesis of entrapment neuropathies has seen a rise in scientific interest over the last decade. In contrast to several recent publications,^8^, ^9^, ^18^, ^21^ by means of an optimised superb microvascular flow (SMI) protocol, we could visualise intraneural vascularity in all examined nerve segments with an average vessel density of 1.21 ± 0.46 vessels per mm^2^ and at least three microvessels per segment. Even though ulnar nerve vascularity within the cubital tunnel was comparably lower, it was still discernible in all cases. Additionally, vendor‐provided vascularity quantification did not correlate with manual vessel count. Several factors were associated with an increase in vessel density, yet at varying degrees of impact. The strongest predictor with a positive correlation was right‐handedness. Sex, height, weight and BMI showed no significant influence.

An extensive network of intraneural microvasculature supplies nerve fibres with the necessary nutrients and energy.^13^ Arterial branches from proximal vessels reach the neural surface and branch into epineural vessels, which penetrate deeper into the nerve forming a network of capillaries at the level of the endoneurium,^13^ while nerve vascularity remains an elusive topic in routine diagnostic examinations of peripheral neuropathies due to technical limitations. We show that through novel ultrasound techniques such as SMI, part of this peri‐ and intraneural vascular network can be visualised. In itself, these findings are not surprising, yet bear implications for the differentiation between ‘normal’ and ‘pathologic’ findings if based on neural vascular changes. In contrast to several recent studies,^8^, ^9^, ^18^, ^21^ where discernible vascularity was often linked to pathological changes in the nerve (as in compression neuropathies) and non‐detectable vascularity to healthy or unaffected nerve segments, our findings underline the ability of special ultrasound techniques such as SMI to visualise intraneural microvessels even in healthy subjects and across relevant nerve segments. Among those studies, Chen *et al*.^8^ who evaluated the blood flow by a simple scoring system (with ‘0 score’ no blood flow, ‘1 score’ one–two spot blood flow, ‘2 score’ two or more spot blood flow or one–two strip blood flow longer than 1 mm and ‘3 score’ more than two strip blood flow), reported that even though 45 of 50 CTS patients showed measurable intraneural vascularisation, 13 of 25 healthy subjects did not. Karahan *et al*.^9^ who used a similar scoring system like Chen *et al*. (‘grade 0’ no vascularity, ‘grade 1’ 1 or 2 focal colour‐encoded spots in the median nerve, ‘grade 2’ one linear colour‐encoded line and more than two focal colour‐encoded spots and ‘grade 3’ more than one linear colour‐encoded line), demonstrated increased intraneural blood flow in cases with severe CTS compared with mild and moderate forms. Interestingly here, five patients with mild and three patients with moderate CTS showed no measurable intraneural vascularity.

In contrast to the results of other studies on ulnar intraneural vascularisation at the cubital tunnel, our findings were comparable to the above mentioned findings in the median nerve with regard to the visibility of blood flow not only in symptomatic patients but also in healthy subjects. While Frijlink *et al*.^22^ showed an increased intraneural vascularisation in 21 of 137 patients with ulnar neuropathy at the elbow, none of the 70 healthy control subjects showed discernible intraneural vascularisation. Similar results were published by Ghanei *et al*.^19^ (27 of 41 patients with cubital tunnel syndrome and three of 44 healthy controls) and Cheng *et al*.^20^ (31 of 67 patients and six of 50 healthy controls). Apart from potential technical differences, one explanation may be the patients' arm flexion during examination (reported at 70°).^8^, ^22^ We preferred a lesser flexion in the elbow of <45° to reduce nerve strain and compression due to surrounding bony and tendinous structures, as higher flexion is known to cause significantly higher pressure in the cubital tunnel.^23^

To our knowledge, there are no recent ultrasound studies on the intraneural vascularity of the common peroneal nerve. As described in an anatomical study by Kadiyala *et al*.^24^ the common peroneal nerve is only sparsely vascularised around the fibular head and neck, which may explain the nerve's susceptibility to and delayed recovery from neural injury. Our findings in healthy subjects do not reflect the clinically evident fragility of the peroneal nerve's vascular bed around the fibular head and neck, but may serve as a baseline finding in cases with delayed recovery after trauma.

The equipment used may factor in the lack of detectable intraneural vascularity in several comparable studies. The reported transducer frequency ranges of 18 MHz (Chen *et al*.^8^ and Frijlink *et al*.^22^), 14 MHz (Karahan *et al*.^9^), 5–7 MHz (Ghanei *et al*.^19^) and 7–15 MHz (Cheng *et al*.^20^) were all lower than the higher resolution 22‐MHz we used. A comparison with the study by Sergeant *et al*. is difficult, as the study focussed on perineural rather than intraneural vessels and no information on the transducer type was provided.

Furthermore, compared with the median and common peroneal nerve, the ulnar nerve within the cubital tunnels showed a decreased relative intraneural vessel density. The vascular supply of the comparably mobile ulnar nerve around the elbow may be influenced by motion‐dependent strain and compression, as the cubital tunnel pressure becomes significantly higher in flexion^23^ and a pre‐existing increase in fibrotic content may compromise vascularity. As Li *et al*.^25^ showed, the main feeding vessel of the ulnar nerve at the elbow is the posterior ulnar recurrent artery, which forms anastomoses with branches of the inferior ulnar collateral artery and/or the superior ulnar collateral artery along the nerve. Therefore, within the cubital tunnel itself, there are mostly only tiny anastomoses^25^ with an overall potentially fragile arterial blood supply, which would explain the decreased intraneural vascularity and its susceptibility to compression‐like alterations in the absence of clear compressing culprits along the cubital tunnel and higher post‐surgical complication rates.^26^, ^27^, ^28^ Even if the mechanical irritation may be remedied, scarring and an abnormal nerve course may further strain an already scarcely vascularised nerve segment, leading to further hypoperfusion.

### Limitations

**Figure 4:**
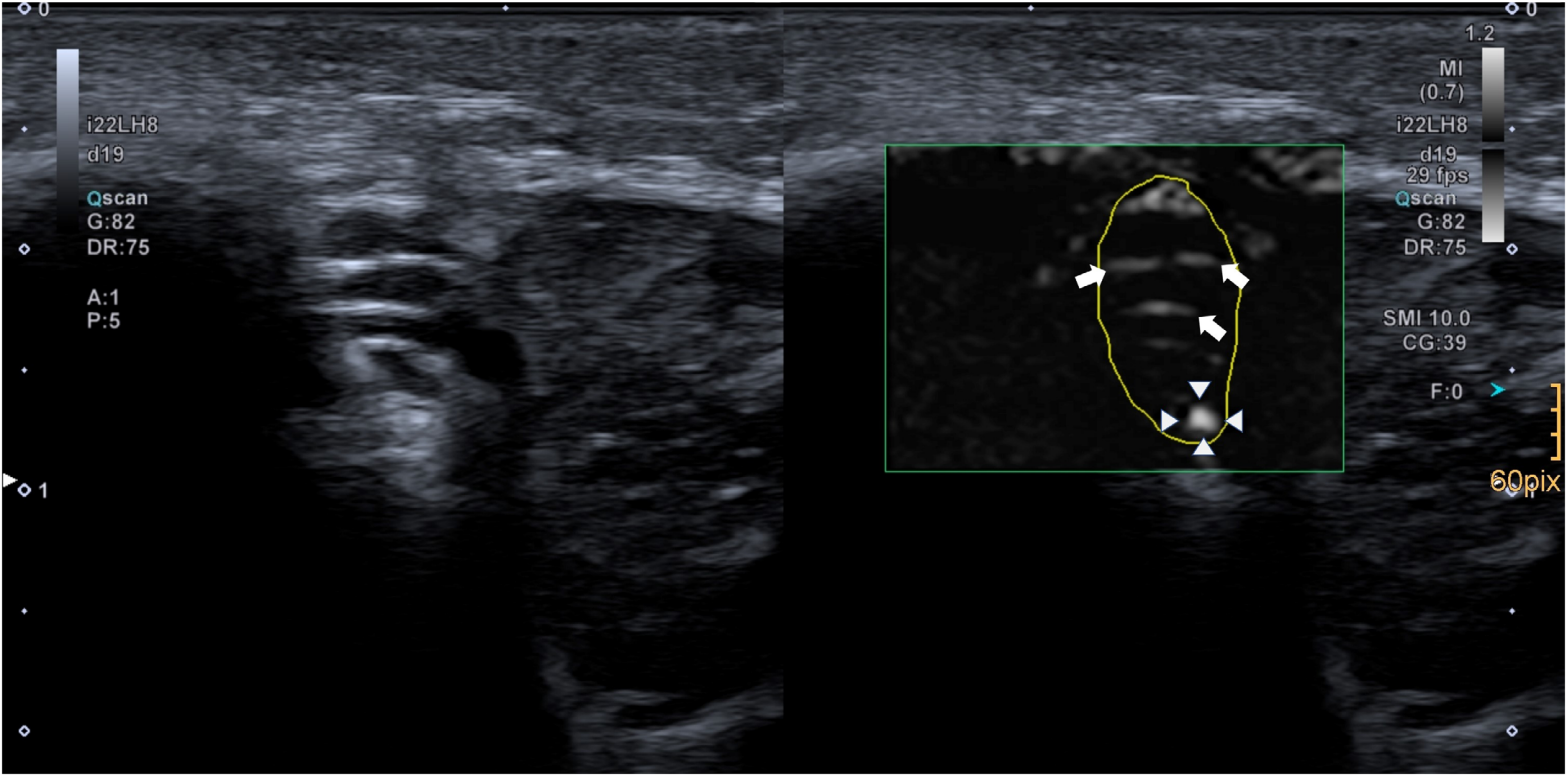
Illustration of intraneural vascularisation of an ulnar nerve with prominent intraneural septa. Intraneural septa echoes may influence the automatically calculated vendor‐specific ‘vascular index’.

A limitation of this study is the missing correlation between the automatically calculated vascular index by the vendor and the manually calculated vessel density. While manual counting is time‐consuming, we estimate that this method delivered the results closest to reality and enables ruling out any false‐positive counts by identifying non‐vascular hyperechoic signals (septa and artefacts) on cine‐loops (Figure 4). Still, this study was not aimed at evaluating the diagnostic power of either. Studies using this simple metric may be misleading in their interpretation of results, though, and researchers should be aware of the automatic method's potential shortcoming. Other limitations included the small subject group with 26 subjects. Furthermore, there may be an influence of limb positioning and transducer pressure on the apparent number of intraneural microvessels. Even though a high degree of standardisation was aimed for during measurements, the true vessel count may be higher in settings where an even greater minimisation of external forces can be achieved.

## Conclusion

Nerve segment vascularity can be visualised by employing high‐resolution equipment and highly sensitive Doppler‐derived methods such as SMI in relevant segments of the median, ulnar and common peroneal nerve in healthy subjects. Thus, researchers and clinicians should not base their definition of healthy nerve segments on the absence of visible vasculature compared with hypervascularity in, for example, compression neuropathy.

Lower neural vessel density encountered in some nerve segments, especially the ulnar nerve, may be a predisposing factor for compression neuropathy.

## Declarations

The authors have no declarations to make.

## Authorship statement

We acknowledge that the authorship listing conforms with the journal's authorship policy and that all authors are in agreement with the content of the submitted manuscript. All authors have consented to the manuscript's publication. All authors contributed significantly in the areas of study design, study planning and conduct, data acquisition, statistics, manuscript drafting and revisions.

## Funding

No funding information is provided.

## Conflicts of interest

None of the authors has any conflict of interest to disclose.
